# Moving GIS Research Indoors: Spatiotemporal Analysis of Agricultural Animals

**DOI:** 10.1371/journal.pone.0104002

**Published:** 2014-08-06

**Authors:** Courtney L. Daigle, Debasmit Banerjee, Robert A. Montgomery, Subir Biswas, Janice M. Siegford

**Affiliations:** 1 Department of Animal Science, Michigan State University, East Lansing, Michigan, United States of America; 2 Department of Electrical and Computer Engineering, Michigan State University, East Lansing, Michigan, United States of America; 3 Department of Fisheries and Wildlife, Michigan State University, East Lansing, Michigan, United States of America; Institut Pluridisciplinaire Hubert Curien, France

## Abstract

A proof of concept applying wildlife ecology techniques to animal welfare science in intensive agricultural environments was conducted using non-cage laying hens. Studies of wildlife ecology regularly use Geographic Information Systems (GIS) to assess wild animal movement and behavior within environments with relatively unlimited space and finite resources. However, rather than depicting landscapes, a GIS could be developed in animal production environments to provide insight into animal behavior as an indicator of animal welfare. We developed a GIS-based approach for studying agricultural animal behavior in an environment with finite space and unlimited resources. Concurrent data from wireless body-worn location tracking sensor and video-recording systems, which depicted spatially-explicit behavior of hens (135 hens/room) in two identical indoor enclosures, were collected. The spatial configuration of specific hen behaviors, variation in home range patterns, and variation in home range overlap show that individual hens respond to the same environment differently. Such information could catalyze management practice adjustments (e.g., modifying feeder design and/or location). Genetically-similar hens exhibited diverse behavioral and spatial patterns via a proof of concept approach enabling detailed examinations of individual non-cage laying hen behavior and welfare.

## Introduction

Many of the fundamental concepts of animal welfare derive from efforts to understand the response of individual animals to prevailing circumstances, and most theories of welfare emphasize a holistic representation of the animal’s experience that encompasses such elements as emotional state, physical health, and ability to perform natural behaviors. Thus, the physical and psychological well-being of animals are frequently examined by assessing behavior, health, and emotional states in a single study, which can be complex and time consuming and require multidisciplinary expertise and invasive measurement. Fortunately, correlations between the behavior of the animal and its internal physiology and emotional state have been demonstrated [1]. Therefore, studying behavior can be a simple and non-invasive way of assessing an animal’s current welfare state.

Individual animals alone, not aggregated groups of animals, experience the characteristics that make welfare better or worse, such as emotional states and health. Thereby, the concept of animal welfare inherently emphasizes the importance of individual experience and variation [2]–[4]. Additionally, individual variation with regards to genetics, experiences, and temperament can impact how an individual animal perceives its current situation, the choices it makes, and ultimately its welfare. However, issues of practicality limit the ability of welfare researchers to gather behavioral data for individual farm animals, because most livestock and poultry in modern production systems are housed in large groups. Further, welfare assessments that include behavioral measures require lengthy observation periods, which are often impractical. Because of these considerations, animal welfare tends to be assessed at an aggregate level and behavioral measures are often employed in a limited or indirect fashion. However, the condition of individuals within a group of animals may vary widely, thus group-level averages may not accurately reflect the condition of specific individual animals within that group. Thus, by using the average condition, researchers may be ignoring those individuals in very poor or very good welfare condition. Combined, these issues highlight the need to develop a method for gathering behavioral information at the level of the individual and interpreting the meaning of this behavior with regard to the welfare of the animal.

Currently, social pressure and political mandates are pushing changes in animal agriculture that are resulting in animals being housed in even larger groups and environments, with the goal of improving welfare and allowing animals more behavioral freedom. For example, the egg industry is transitioning from housing laying hens in groups of 4–10 hens in small, simple cages to housing hens in groups of hundreds or thousands in large (mostly) indoor enclosures that contain a variety of environmental features such as nest boxes and perches. The hens housed in these emerging systems likely face different welfare challenges than conventionally-caged hens, which must be recognized and addressed to ensure that alternative housing systems do in fact improve welfare. Yet current assessments only describe group welfare, possibly masking individual problems.

Information on how individual non-cage laying hens use space and the spatio-temporal variation in their space use is poorly understood. This dearth of information results from the methodological challenges inherent to describing individual animal behavior in group settings. By implementing technological advancements (e.g., wireless sensors) to a representative proportion of the population and adopting analytical techniques used by other disciplines (e.g., Geographic Information Systems (GIS)), we may be able to better capture the responses of individuals housed in large groups. Modeling the spatial configuration of hen behaviors can provide insight into the general welfare of the individual. For example, feeding (here defined as consuming food from a feeder) and foraging (i.e., searching for and/or consuming food found in litter using the feet and beak) are both behaviors that hens are motivated to perform and are required for survival [5], [6]. Preening (i.e., a maintenance and comfort behavior where feathers are cleaned with the beak) can be considered a comfort behavior and has been observed to be performed more often in the presence of familiar conspecifics [7]. However, based on previous studies [8], [9], it is clear that hens make different choices with regard to where and when they choose to perform these welfare-relevant behaviors based upon their perception of the environment.

Hen behavior is changing in alternative housing systems, compared to what was expected in conventional systems and these changes must be recognized so that management practices and physical housing designs can also be changed to complement the behavioral changes in ways that improve hen welfare or maintain it at a high level. Furthermore, the application of this technology would allow animal managers to identify what should be expected, and this approach would provide them with a new tool to monitor flock movement and identify events when changes in behavior could indicate welfare concerns (e.g., increased activity levels associated with the development of feather pecking [10]). Production animal systems are beginning to mirror some of the large group phenomena that are observed of animals found in natural environments, such as colonial breeding groups of sea birds that face competition for space and resources due to the high concentration of animals in a single area [11]. Thus, management practices and scientific approaches should begin to mirror those applied in wildlife behavioral ecology, as laying hens housed in large groups with limited space and finite space to access resources may face similar challenges to wild species.

Here, we examined spatial patterns in hen behavior and movement in an indoor agricultural setting by combining wireless sensor technology with GIS. Via these techniques, we provide the first characterization of individual hen home ranges in a non-cage environment and the first illustration of spatiotemporal variability in individual hen behavior. This analysis represents the first effort, to our knowledge, to combine wildlife ecology techniques and wireless sensor technology in the study of animal behavior in an intensive indoor, animal production environment.

## Methods

### Animals and housing

**Figure 1:**
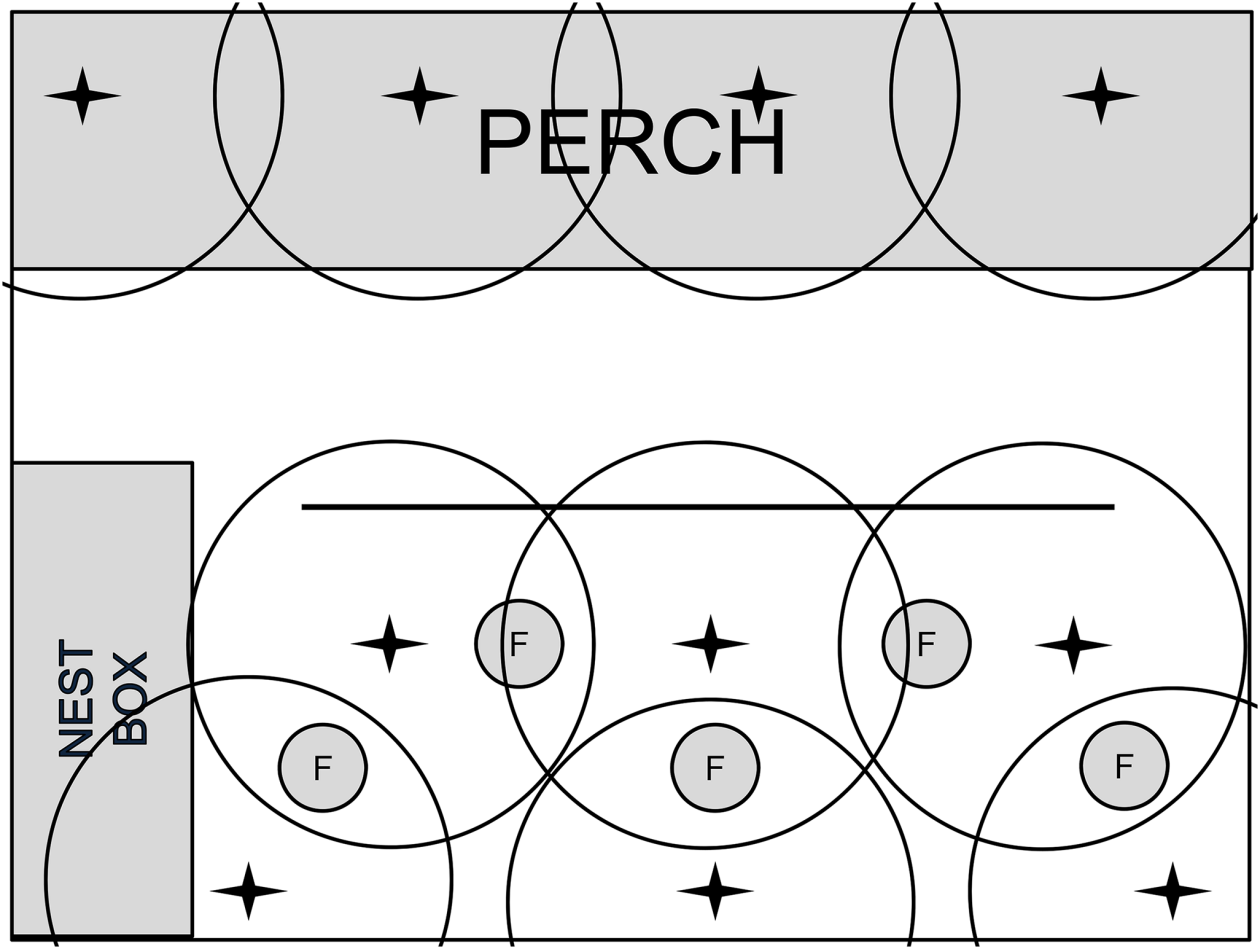
Diagram of room set up and stationary node placement. F represents a feeder. The water line is represented by the thick solid line in the middle of the room.

We collected data from laying hens housed in an experimental non-cage system at the Michigan State University Poultry Teaching and Research Center (MSU-PTRC). Prior to the start of the study, all protocols were submitted to and approved by the Michigan State University Institutional Animal Care and Use Committee (AUF: 04/08-060-00). Two identical rooms (6 m×4.5 m) at MSU-PTRC were used. Each room was furnished in the same configuration with nest boxes, perches, tube feeders, and a water line with nipples (Fig. 1). Sixteen nest boxes (each 0.4 m long×0.3 m wide×0.3 m high) in an 8×2 configuration were mounted 0.3 m above the ground on one wall. Perches consisted of a three-level wooden rail structure (with each rail 6 m long and ∼5 cm in diameter with a flat top and rounded sides and bottom) and mounted over a 1 m×6 m slatted area at a height of 0.53, 0.76, and 0.99 m from the ground. The perches were mounted to the wall at a slope of 45° with a 40 cm distance between each wooden rail. Room floors were covered with ∼8 cm of wood shavings at time of data collection. Food and water were provided daily ad libitum. Daily care, including egg collection, feeding, and hen inspection, occurred at least once a day. Two incandescent light bulbs (60 lux at bulb level) on an automatic timer provided light 15 h per day in each room. Temperature was maintained between 16°C–22°C using a ventilation fan and forced air heating.

Hy-Line Brown laying hen pullets (n = 270, 135 hens/room) were reared in each of the rooms as described above with accommodation made for their size (i.e., smaller perches, which were removed at 6 weeks of age (wk)) and immaturity (i.e., access to nest boxes was not granted until 10 wk of age). Each room provided 0.21 m^2^ floor space, 17.8 cm of perch space, 0.01 m^2^ of nest box space, and 4.83 cm feeder space per hen (Fig. 1). Thirteen nipples provided drinking access at a ratio of 10.3 hens per nipple. All room parameters met or exceeded United Egg Producers and the Federation for Animal Science Society’s housing requirements for non-cage laying hens [12].

**Figure 2:**
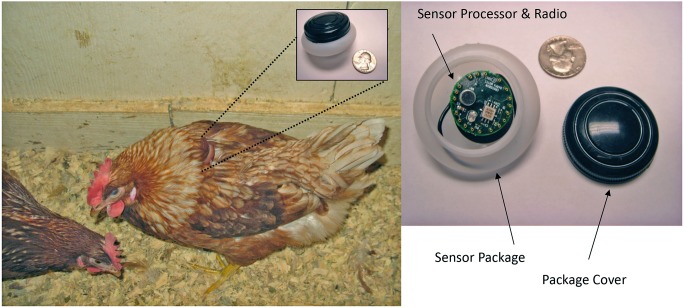
A laying hen wearing a wireless sensor. The wireless sensor is packaged in a plastic case to prevent entry of dust and moisture. We mounted the sensor the on the back of the hen using a figure-eight nylon harness. Both the sensor case and the harness were colored to blend in with the hen’s feathers to avoid attracting the attention of other hens in the experimental room. This picture was taken several days after the hen was fitted with the sensor.

**Figure 3:**
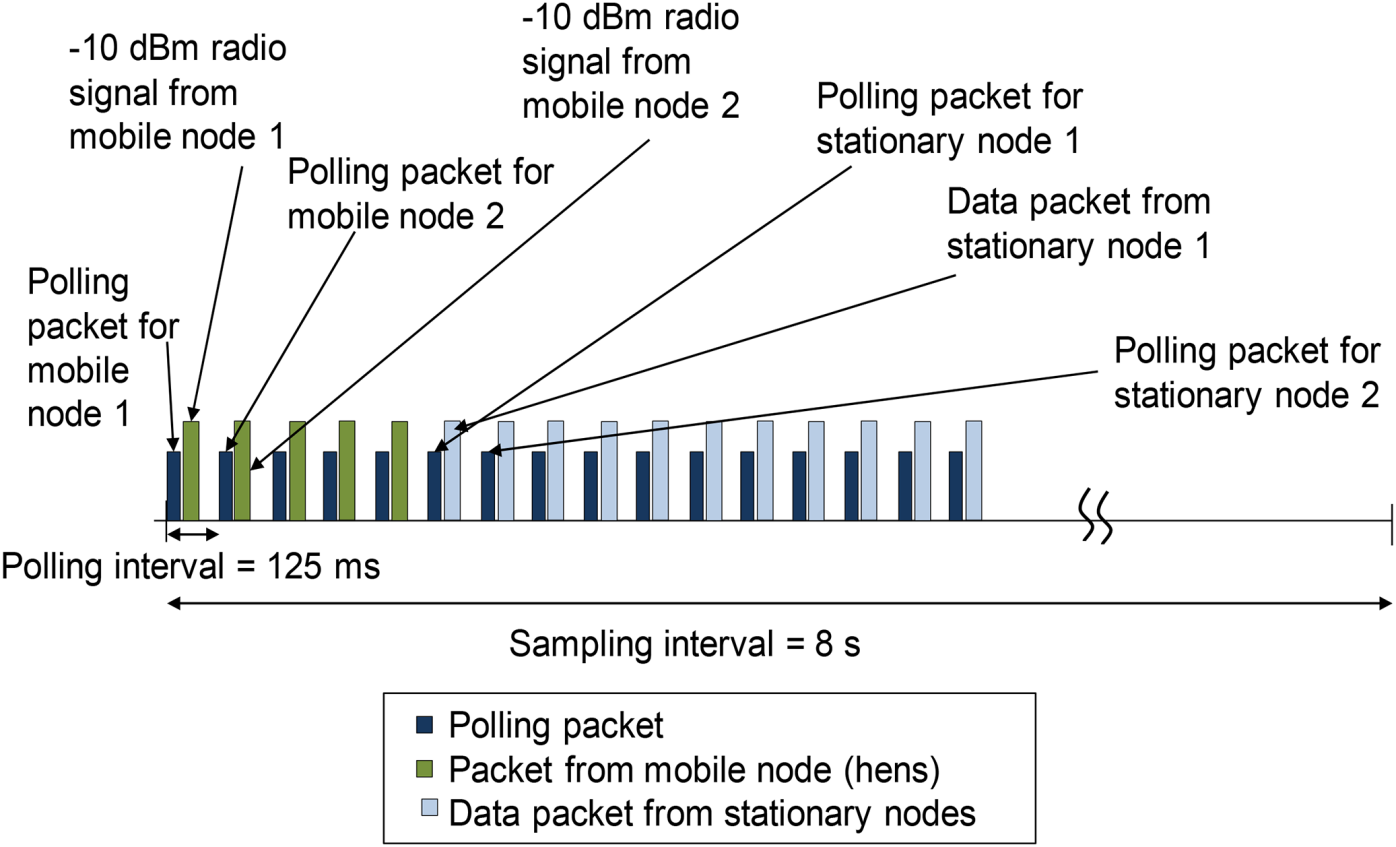
Illustration of the polling-based time division multiple access (TDMA) protocol.

At 10 wk, we weighed all hens and fitted them with uniquely numbered leg bands. At 11 wk, 10 hens per room were selected and fitted with a sensor (Fig. 2). We selected hens from different part of the spectrum of body weights because body weight contributes to hen social hierarchy. For instance, heavier hens are more likely to have won a recent fight and perform more double attacks when establishing a social hierarchy [13]. Thus, this step enabled us to evaluate behavior of hens from a variety of social ranks. Collection of data from the sensor to monitor the hen’s behavior prior to and immediately after the onset of lay (approximately 18 wk) as part of a separate experiment was the motivation for fitting the hens with sensors prior to comb development. Therefore, even though combs are important to hen social hierarchy [14], it was not possible to include comb size as a parameter indicative of social status to select hens to wear sensors. The remaining 125 hens in each room did not receive sensors. Previous research indicated that wearing the sensor did not have any long-lasting effects on hen resource use or agonistic interactions [8]. The sensor has been shown to not impact hens’ resource use or agonistic interaction [15] and can collect locational data (i.e., proximity to stationary sensor) from multiple hens simultaneously through a time division multiple polling access (TDMA) protocol as illustrated in Figure 3. One individual died between the two data collection points, so the final analysis includes data from nine individual hens.

### Wireless sensor network

We attached a mobile sensor to each of nine individual hens housed in two separate, identical rooms (five hens in one room, four in the other) using a nylon figure-eight harness. Each mobile node (∼10 g) was placed inside a plastic casing and mounted on a hen’s back with figure eight nylon harness. The casing was colored to match feather color and painted with a unique number for easy visual identification (Fig. 2). After experimentation, mounting the sensor on the back of the hen was determined to yield maximum sensor stability while maintaining sufficient signal quality for proper wireless communication with stationary nodes and avoiding tissue damage to the hen. These same hens wore the sensor casing throughout the lay cycle. The base station was strategically placed outside of the room to maintain communication with all stationary nodes. The mobile sensors communicated with a network of stationary sensors strategically placed throughout the hens’ environment (Fig. 1).

The sensor system consisted of three components – (i) a Mica2Dot mote radio mobile mounted on hens, (ii) Mica2 mote stationary radio nodes acting as beacons for the proximity detection process, and (iii) a base station that collected the data wirelessly from stationary nodes and stored the data in a laptop PC. Each component consisted of a processor and a radio subsystem, running a Tinyos operating system. All network components operated via a 900 MHz wireless radio link. We strategically placed the ten stationary nodes throughout the room (Fig. 1). To minimize communication interference between stationary and mobile nodes, the stationary nodes were hung one meter above the ground with ceiling-mounted PVC pipes to point their antennas downwards. A polling-based time division multiple access (TDMA) protocol (Fig. 3) administered communication between the nodes in the wireless network at a rate of 0.125 Hz (8 sec sampling interval) to maximize battery life and maintain network synchronization. Furthermore, we implemented an energy-aware sleep schedule to minimize battery consumption and extend the mobile node operating life which allowed the system to run for 48–50 h.

### Behavioral data collection

We video-monitored hens in each room continuously across 48-h intervals at 48 and 66 wk using ceiling mounted cameras. The detection of behavior using video was necessary because technology development to detect hen location and behavior simultaneously is in the nascent stages [16]. By associating these observations with the output of the sensor network we made our behavioral data spatially explicit. For instance, observations of feeding, foraging, and preening collected from video were made spatially explicit by time-syncing them with locational data produced from this sensor network. Though 10 hens per room were fitted with sensors, video data were only decoded for the five individual hens per room that were actively transmitting sensor data, as five mobile nodes was the maximum the sensor network could currently poll at that stage of development and retain network synchronization. Therefore, the individual behavioral data collection for this research was restricted to the same five individuals per room.

Continuous observation of individual hen posture, behavior, and resource use (Table 1) was made over a 30 min period every hour and a half (06∶00–06∶30, 07∶30–08∶00, 09∶00–09∶30, 10∶30–11∶00, 12∶00–12∶30, 13∶30–14∶00, 15∶00–15∶30, 16∶30–17∶00, 18∶00–18∶30, and 19∶30–20∶00). Data were collected only during the lights on period following a validated sub-sampling procedure for hens housed in this way and the specific behaviors of interest [17]. Data were collected across a 48 h time period with the dark period (21∶00–06∶00) omitted because even though infrared cameras were utilized to observe night time behavior, substantial amounts of movement were not observed (most movement during lights off was due to hens transitioning between standing and sitting as they readjusted during the night). Therefore data collection efforts were focused on the lights on period only. Posture, behavior, and resource use were recorded in mutually exclusive categories and are reported in duration of time spent (sec) in that state across the 48 h period and paired t-tests (PROC TTEST, SAS version 9.2) were employed to identify statistical differences between the two ages in the amount of time performing each behavior.

**Table 1:** Ethogram of behaviors developed to identify posture, behavior and resource use.

*Posture*	Description
Walk	Walking more than 3 steps in succession with head up or when walking hen has not been standing, drinking, feeding, or foraging for the previous 5 s
Stand	Hen is upright and supported off of the ground or perch by legs
Sit	Hen is upright with body touching the ground or perch
***Behavior***	
feed	Hen pecks at feed in the feeder. Recording starts at first peck
drink	Head is turned upwards towards water source, and hen uses beak to peck at one of the nipples, apparently consuming water
preen	Hen may be sitting or standing. Beak is used to manipulate, rearrange, pull, or smooth body feathers on self. Beak is often run along the length of the feather, starting at the base and moving out towards the tip of the feather
dust bathe	While squatting or laying, hen performs dust bathing activities including vertical wing shaking, bill raking, scratching, ground pecking, movements of the feet and wings to raise dust into the ruffled plumage, rubbing of head and sides in the dust, feather-ruffling and shaking dust out of the feathers. Starts with first wing shake
forage	Hen pecks at substrate while standing or stepping forward with head below rump level. Starts when the hen makes >3 successive pecks at substrate, or when foraging hen has not been standing or walking with head up, or feeding, for the previous 5 s
rest	Starts the hen lies down (sternum resting on substrate) from an upright position or when lying bird has made no dust bathing or preening movements for the previous 8 s
***Resource***	
feeder	Hen has head in feeder and pecks at feed in the feeder
drinker	Head is turned upwards towards water source, and hen uses beak to peck at one of the nipples, apparently consuming water
perch	Hen is standing, walking, or resting on perch, the rail in front of the nest boxes, or black base of slats underneath raised perches
nest box	Hen is standing or resting inside a nest box

A hen was considered to be performing a new posture, behavior, or resource use when she stopped performing the previous behavior for >5 s or began performing a new behavior for >5 s.

### Combining sensor network output and behavioral observations into GIS

We used the spatially-explicit behavioral data to depict hen home range distributions and the spatial distribution of welfare-revealing behaviors. In this respect, we focused our assessment on feeding, foraging, and preening behaviors which can be associated with physical health (e.g., nutrient acquisition or foot condition), natural behavior expression (e.g., grooming and maintenance behaviors) and emotional state (e.g., frustration due to hunger or comfort due to the presence of familiar conspecifics).

Expression of home range and behavior across two periods (48 and 66 wks) allowed us to examine whether these processes varied by individual hen and across time. Specifically, data depicted home range patterns within the non-cage environment, identified the size and amount of overlap in home ranges of hens within these enclosures, and illustrated whether spatial segregation by behavior was apparent among the hens [18]–[20].

To assess this spatiotemporal variation we needed to develop a GIS depicting physical properties of the indoor enclosures. We georectified and virtually represented the exact dimensions of the room where hens were housed in ArcMap 10.0 (Environmental Systems Research Institute, Redlands, CA). Gaps in the sensor network required that probabilistic movement paths were interpolated between missing locations for each individual hen. Therefore, we fitted a continuous-time correlated random walk model to the sensor data [21]. We developed this model in R (R statistical software version 2.15.1, <www.cran.r-project.org>, accessed 1 May 2013) using the package CRAWL which employed a Kalman-Filter to predict locations from the existing point pattern based on a continuous-time stochastic movement process. In this way, locations that were missed because of gaps in the sensor network were imputed to make the database of hen locations complete.

We used this locational database to delineate home ranges for each individual hen in both rooms by time period (48 and 66 wk). Utilization distributions, widely used in the wildlife sciences, depict the relative probability of animal occurrence in space. In this capacity, we developed utilization distributions (UDs) [22] in R using the Kernel Density Estimator library. We fitted these UDs using a bivariate plug-in matrix that calculated bandwidth along rotated axes for each individual hen [19], [20], [22]. We subsequently calculated the amount of overlap among the hen UDs for each time period.

To map the spatial configuration of hen behaviors, we developed hotspot maps depicting foraging, preening, and feeding behaviors. Thiessen polygons, which are well-suited to binary data [18] (i.e., the presence or absence of the behavior of interest), were utilized to develop the spatially-explicit maps of these behaviors. The precision of the sensor network (8-sec intervals) meant that hens were recorded performing multiple behaviors in the same location. Thus, the Thiessen polygons present the mean count of the behavior of interest within each individual’s home range. For presentation purposes, these data are divided into equal intervals representing space use for foraging, preening, and feeding in increments of high, medium, low, and none, which correspond to whether the animal was observed performing the behavior more than 75% of the time (high), 74–25% of the time (medium), less than 25% of the time, or not at all (none).

## Results

### Behavioral observations

Hens varied in the amount of time spent performing specific behaviors (Table 2), and paired t-test determined that hens tended to spend a smaller proportion of their time feeding at 66 wk compared to 48 wk (t_8_ = 1.91, P = 0.09). No significant differences were observed the percentage of time the hens spent performing the rest of the behaviors between the two ages.

**Table 2:** Percent of time spent performing behaviors for hens at 48 and 66 weeks of age.

Hen ID	Age	Feed	Drink	Preen	Dust bathe	Forage	Rest	Other
B01	48	40.62	0.00	8.59	0.00	16.03	1.78	32.97
	66	22.00	6.48	7.93	2.61	15.62	2.87	42.49
B10	48	12.10	2.69	19.36	0.18	15.47	1.98	48.23
	66	2.91	3.42	1.44	0.00	2.79	0.00	89.44
B02	48	31.46	1.85	12.15	2.73	9.51	11.09	31.21
	66	17.07	3.87	12.91	1.69	17.48	1.95	45.04
B05	48	32.35	4.92	9.18	2.32	15.11	4.22	31.90
	66	20.77	3.79	9.66	1.33	17.03	1.93	45.49
B08	48	1.71	3.54	19.82	2.63	22.45	14.14	35.71
	66	0.45	3.09	15.39	2.18	27.53	0.00	51.36
Y10	48	17.28	4.27	7.72	0.00	20.77	6.55	43.40
	66	10.41	6.22	21.36	4.22	19.14	4.89	33.77
Y05	48	18.45	2.19	16.78	1.80	17.68	0.75	42.34
	66	24.10	2.46	16.02	4.31	8.00	4.06	41.05
Y07	48	0.09	1.44	5.45	0.00	0.00	0.00	93.02
	66	0.03	1.33	2.58	0.00	0.00	0.00	96.06
Y08	48	1.33	2.68	18.75	0.24	30.05	3.39	43.57
	66	7.46	2.27	23.29	0.00	19.01	11.55	36.42
Average	48	17.26	2.62	13.09	1.10	16.34	4.88	44.71
SEM		4.99	0.50	1.88	0.41	2.80	1.62	6.37
Average	66	11.69	3.66	12.29	1.82	14.07	3.03	53.46
SEM		3.19	0.57	2.54	0.56	2.92	1.22	7.64

Results are presented for hens from two separate rooms (Blue, as indicated with a B and Yellow as indicated with a Y) at two different ages (48 and 66 wk).

### Hen home ranges and conspecific overlap

**Figure 4:**
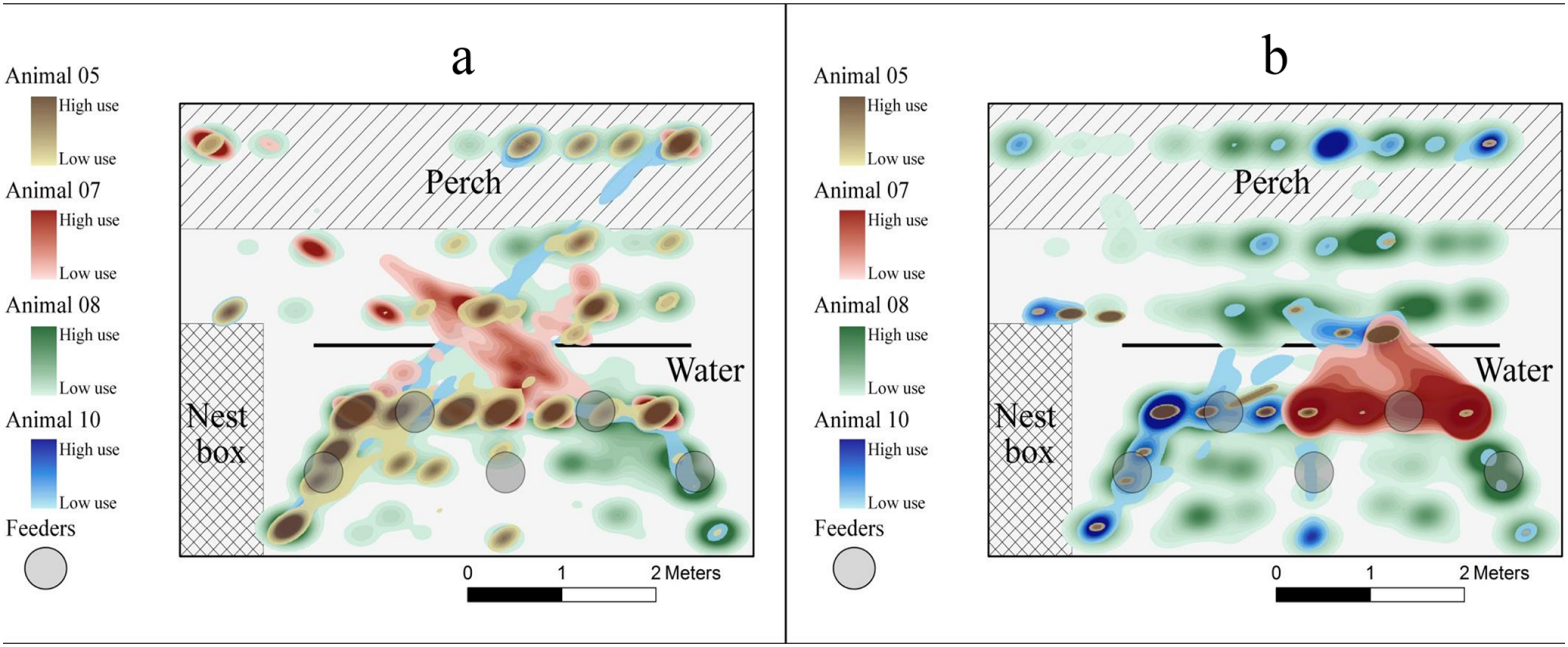
Overall utilization distribution for hens housed within Room Y at 48 (a) and 66 (b) wk. The utilization distribution for Room B is similar and shows a similar amount of variation among hens and across time. Room B is not pictured here due to clarity of image in black and white.

Hens exhibited large differences in home range size and amount of overlap with conspecifics (Fig. 4). On average, home range size (m^2^) at 48 wk was larger (5.48±1.13) than at 66 wk (5.17±1.48), while the proportion of overlap (%) with conspecifics decreased from 0.88±0.05 at 48 wk to 0.84±0.07 at 66 wk. Hens that used a smaller proportion of the room were observed to have more of their home range overlap with their conspecifics (i.e., Table 3, Y05), while hens with larger home ranges had less home range overlap (i.e., Table 3, Y08).

**Table 3:** Hen home ranges and home range overlap at two different ages.

Animal ID	Age (wk)	Range size (m^2^)	Proportion of room used	Proportion of overlap with conspecifics
B01	48	4.82	0.17	0.97
	66	1.65	0.06	1
B02	48	1.52	0.05	1
	66	7.64	0.27	0.94
B05	48	6	0.22	0.89
	66	9.11	0.33	0.84
B08	48	6.83	0.25	0.94
	66	7.68	0.28	0.85
B10	48	11.28	0.41	0.76
	66	1.66	0.06	0.99
Y05	48	2.97	0.11	1
	66	0.37	0.01	1
Y07	48	2.8	0.1	0.9
	66	1.82	0.07	0.76
Y08	48	10.04	0.36	0.5
	66	13.35	0.48	0.27
Y10	48	3.14	0.11	0.96
	66	3.26	0.12	0.9

Home range area (m^2^), proportion of the room that the home range covers (%), and proportion of individual hen’s home range that overlaps with her conspecifics (%). Results are presented for hens from two separate rooms (Blue, as indicated with a B and Yellow as indicated with a Y) at two different ages (48 and 66 wk).

Substantial variation was observed among the hens with regard to overlapping home ranges. Five hens (Table 3, B05, B08, Y07, Y08, and Y10) showed a relative decrease in the amount of overlap as they aged; while three (i.e., Table 3, B01, B2, and B10) showed an increase and one remained relatively consistent (i.e., Table 3, Y05). Consequently, this was reflected in changes observed in pairwise associations (m^2^) describing degree of overlap between pairs of hens (Table 4).

**Table 4:** Total area (m^2^) of overlap between two hens at two different ages (48 and 66 wk).

Pairwise overlap between	48 wk	66 wk
B10–B01	4.39	0.51
B10–B02	1.49	1.53
B10–B05	5.09	1.59
B10–B08	6.21	1.15
B01–B02	1.27	1.64
B01–B05	3.65	1.64
B01–B08	3.10	1.57
B02–B05	1.09	6.57
B02–B08	1.43	5.49
B05–B08	2.98	5.87
Y05–Y07	0.08	1.01
Y05–Y08	0.33	2.87
Y05–Y10	0.03	2.07
Y07–Y08	1.37	2.48
Y07–Y10	0.77	1.22
Y08–Y10	2.88	2.88

### Feeding, foraging, and preening

**Figure 5:**
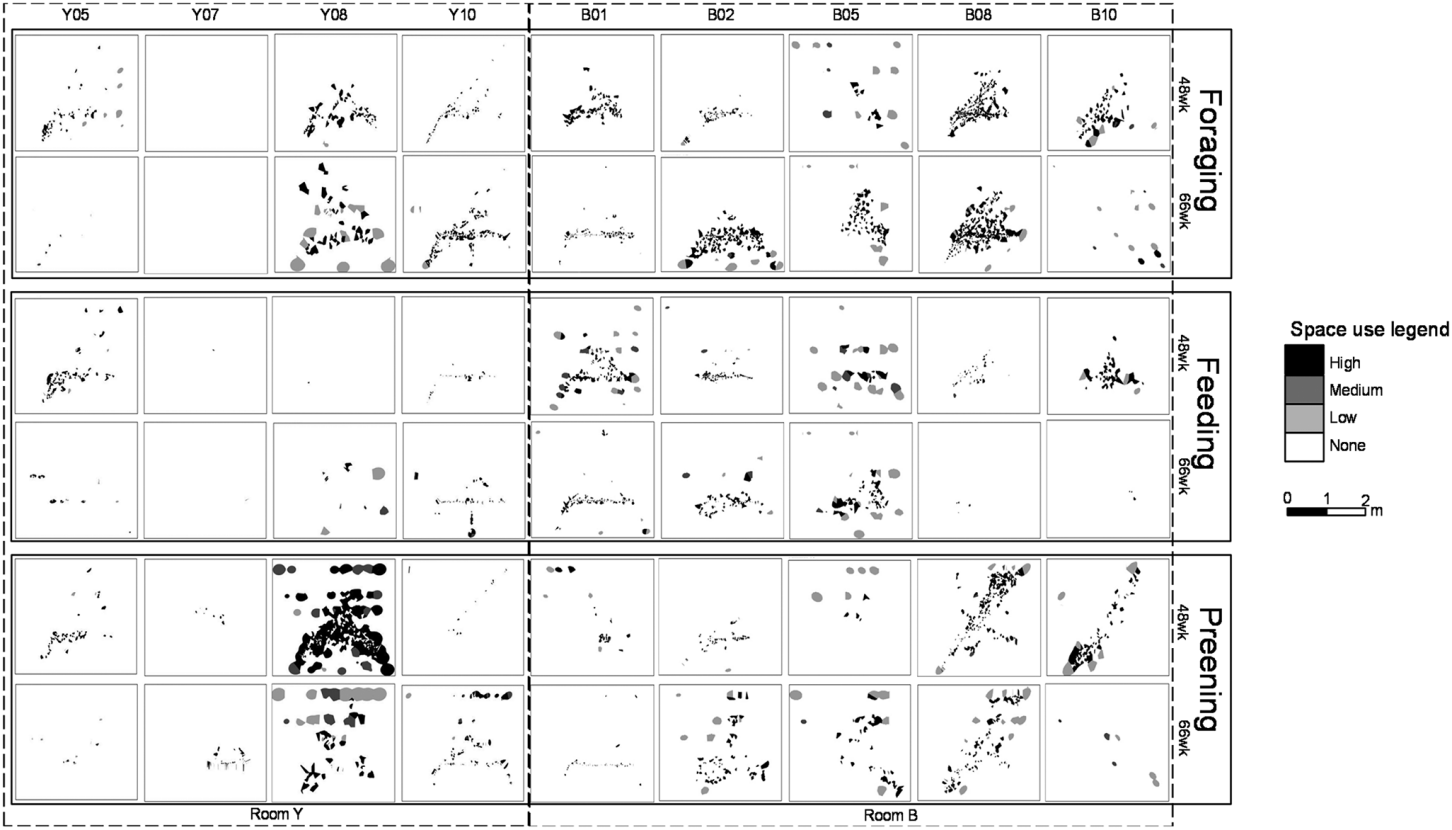
Hotspot maps of feeding, foraging, and preening behavior at 48 and 66 **wk.** The labels at the top of each panel indicate which hen is represented. The legend to the right illustrated the amount of time (high, medium, low, none) the hen spent performing feeding behavior in that area, which correspond to whether the animal was observed performing the behavior more than 75% of the time (high), 74–25% of the time (medium), less than 25% of the time, or not at all (none)”.

We observed a wide variety of feeding, foraging, and preening patterns among the nine hens across time (Fig. 5). Several hens (Y10 and B05) were relatively consistent in the locations they used to perform feeding, foraging and preening. B08 and B10 both performed more foraging than feeding, and B02 appeared to make different choices with regard to foraging and preening at 48 wk compared to 66 wk.

The average percentage of the hens’ time devoted to feeding and foraging decreased with age (Table 2). There was also a wide diversity in how much and where preening occurred. Two hens (B08 and B10) within the same room were observed to exhibit similar preening and foraging patterns across time. Y07 was observed to not spend any time foraging or feeding at either age.

Associations among the amount of time spent feeding, foraging, and preening revealed that the amount of time spent foraging was positively correlated with the amount of time spent preening (R_18_ = 0.653, P = 0.0033), yet was not associated with the amount of time feeding (R_18_ = −0.021, P = 0.935) even though this relationship was negative. A weak, negative relationship was observed between feeding and preening (R_18_ = −0.106, P = 0.676).

## Discussion

Concerns about animal welfare, including the ability of animals in agricultural production systems to perform natural behaviors, demand that tools are developed to better understand individual animal behavior. Here we provide an example of analysis using data obtained from behavioral observations and a wireless sensor network using GIS that could be used to dynamically assess animal welfare. By combining locational data from the sensor with behavioral observations, spatiotemporal differences among individual laying hens in a non-cage environment were identified across time. Furthermore, these results highlight the large degree of individual variation exhibited among laying hens with regard to their overall and behavior-specific space use. Changes in the location and frequency of behaviors could provide insight into individual hen condition, while variability among hens within the same environment can be used to help identify areas important for performance of specific behaviors and evaluate whether space is a limiting factor in the performance of behavior. This information can improve housing design and animal management practices with regard to animal welfare, not only for laying hens, but for other poultry and livestock species, or, in fact, any confined animals, but particularly those managed in large groups.

In the present study, hen movement within the non-cage system was restricted by the confines of the room. When wild animals are observed to have overlapping utilization distributions, these overlaps can identify areas of optimal habitat for reproduction, foraging, or resting. However, it is likely that hens in a closed system are more likely to show overlap for non-traditional reasons. Hens may exhibit more or less fidelity to specific areas of the room based upon personal preferences, or hens may have a high degree of overlap due to social interactions or resource availability. For example, hens may also have a high degree of overlap simply due to the stocking density of the room and have no choice but to inhabit one another’s space. However, further investigation is needed to understand similarities and differences in space use by confined animals compared to wild animals that are free ranging.

Remote monitoring of animals and the development of a GIS can be used to assess the behavior and welfare of agricultural animals in indoor spaces as much as it can for wildlife living in natural environments. In the present study, analysis of home range size, home range overlap, and behavior revealed considerable variability among individual hens. This individual variation provided a window with which we could better view the behavioral differences of certain hens in their enclosures. When we applied this tool to our group of hens, our analysis revealed vast individual variation. Information from such utilization distributions can have implications for hen welfare because they can give insight into where hens are choosing to perform certain behaviors, which may result in altered management practices or facility design. Further, this approach can be used to address multiple types of welfare related concerns across multiple species. For example, future studies applying this approach could identify the impact of sex ratios on the behavior of breeding flocks; the impact of mixing and social dynamics on group-housed sows; or environmental preferences of dairy cattle.

Animal behavior can provide insight into the welfare of the animal. For example, animals behave differently when they are ill (e.g., they may exhibit reduced feeding behavior), injured (e.g., they may exhibit reduced locomotor behaviors), or distressed (e.g., laying hens perform more vocalizations when they are frustrated and unable to perform behaviors such as feeding and drinking [23]). Therefore, changes in their behavioral patterns can be used as a proxy measure for how the animal may be feeling and its overall welfare state. A marked difference in space use was observed for Y07, in which the hen used a smaller proportion of the room at 66 wk compared to 48 wk. The reason for this change in use is unknown, but changes such as this could be indicative of a physical (i.e., the hen may choose to move less due to an injured foot) or psychological issue (i.e., the hen may choose to move less due to an increase in agonistic social interactions). However, for other species different behaviors may be of more interest and provide more insight based upon the biological relevance of the behavior.

In managed conditions, food is only provided at a feeder(s) and the location of feeding behavior is largely restricted to feeder locations. Thus, for hens in production systems, feeding requires access to a highly valued and spatially concentrated resource. Some hens may be restricted from freely accessing the feeder due to social interactions (i.e., competitive exclusion). When multiple feeders are provided, it is unknown whether hens consistently use the same feeder or whether some feeder locations are more attractive than others. In the present study, one hen appeared to perform feeding behavior in a single area (e.g., Y10), while other hens distributed their feeding behavior evenly throughout the room in which feeders were located.

Some hens may choose to not use the feeder, however, acquiring their nutrients from feed that has been spilled on the ground by other hens. These hens may be avoiding the feeder because they prefer to acquire their food via foraging in litter, they may be thwarted from accessing the feeder due to social interactions, or they may forage due to inherent behavioral drives. Based on the results of this study, hens may fall into any one of those categories, as they appear to adopt resource acquisition strategies based upon current physical and/or social constraints, and do not appear to develop preferences for specific feeders or feeder locations. Beyond using such information to better manage and design systems, changes in feeding behavior could be indicative of illness [24], injury [25], [26], or change in social dynamics [27], which could be better studied using the type of data presented here.

However, little is known about the degree to which individual hens may forage versus feed or whether such preferences or patterns change over time. Foraging is not as spatially constrained by the presence or absence or location of specific resources as feeding from a feeder would be, for example. Therefore, the location of hens’ foraging behavior may also help producers identify environmental conditions that are favorable for foraging, such as shavings or straw on the floor or provide insight into the amount of foraging space needed. Two hens (B08 and B10) were observed to use the feeder very little, but spent a large proportion of their time budget foraging. Hens must be mobile to forage, able to move around in the enclosure as they search for and consume food by this method. Therefore, changes in the amount of time spent foraging or in the area in which foraging occurs could be indicative of several health problems including illness, painful foot infections [28], neuromas resulting from improper beak trimming [29], broken claws, or broken bones – all of which are relevant to welfare.

Patterns of preening behavior can provide insight into where hens choose to rest, groom, and spend time with familiar conspecifics, and may thus provide insight into the hen’s mood or emotional state. Preening can also be a response to stress [30] or be performed as a displacement behavior when motivation or behavioral drives of hens are thwarted [31]. Preening can occur at any time of the day and is not constrained by posture (e.g., sitting or standing) or location of the hen in the environment [32]. Therefore, variation in preening behavior may be indicative of differences in health, maintenance, stress response, preference, or comfort among different hens. Thus, future investigations using dynamic modeling could be used to better elucidate social-spatiotemporal relationships.

Here, a proof of concept approach is presented using GIS to understanding the relationship between non-cage laying hen behavior and space use. This approach provides an opportunity to ask many types of questions important to animal welfare in human-managed environments. With this in mind, concessions must be made with regards to needed improvements to the system as research efforts continue. Duration of data collection (approx. 48 hours) was constrained by battery life. Future research efforts should address how the kinetic movement of the animal can power the technologies used to track their movement and provide welfare-relevant information to the producer and consumer. Data were collected from only five hens per room. As this type of analysis becomes more commonplace, it will be important to identify an optimal sample size for large groups of animals that can provide information important to their management. Also, implementing this technology currently requires expertise in computer programming and data analysis. A generalized user interface is under development for the sensor system used in this study, and implementation of this interface would make the collection and analysis of data less time consuming and more accessible.

Commercially-housed laying hens have been genetically selected to be similar with regards to growth, nutritional needs, productivity, and longevity. Despite this genetic similarity, and even despite identical rearing environments, the mature hens housed within the same environment in this study exhibited very different patterns of space use and had home ranges of varying sizes. Furthermore, some hens exhibited changes in their behavioral patterns over time, while others remained relatively consistent – which may be reflective of different levels of behavioral plasticity or changes in social status or health as the hens aged. This work emphasizes that even though hens may be genetically similar, they still exhibit individual behavioral patterns, meaning that designing housing or management practices for the ‘average’ animal will not ensure good welfare for all individuals in the system. Therefore, there is a need to provide environmental conditions that can accommodate the diverse spatial needs and preferences for all of the hens housed within. Understanding the diversity of behavioral patterns will enable animal managers to develop best practices and provide resources so animals of all social ranks, personalities, and preferences can acquire the necessary resources and perform the necessary behaviors indicative of a positive welfare state.
